# Perianal leiomyosarcoma as a rare sequela of rectal cancer radiotherapy: a case report

**DOI:** 10.3389/fonc.2024.1474536

**Published:** 2024-11-04

**Authors:** Chaopeng Chen, Wenping Cai, Yujiao Li, Junqi Ren, Zhibin Xu, Lijuan Pang, Weiping Dai

**Affiliations:** ^1^ Department of Pathology, Central Hospital of Guangdong Provincial Nongken, Zhanjiang, Guangdong, China; ^2^ Department of Pathology, The Central People’s Hospital of Zhanjiang (Zhanjiang Central Hospital, Guangdong Medical University), Zhanjiang, Guangdong, China; ^3^ Department of Pathology, The Second Affiliated Hospital of Guangdong Medical University, Zhanjiang, Guangdong, China; ^4^ Department of Organ Transplantation, The First Affiliated Hospital of Guangzhou Medical University, Guangzhou, Guangdong, China

**Keywords:** radiation-induced sarcoma (RIS), rectal adenocarcinoma, leiomyosarcoma, neoadjuvant chemoradiotherapy, pathological diagnosis

## Abstract

Radiation-induced sarcomas (RIS) are iatrogenic malignancies that arise following high-dose radiotherapy, posing a significant clinical challenge due to their poor prognosis and resistance to conventional treatments. The incidence of RIS is increasing with advancements in radiotherapy techniques. This report presents a case of a 71-year-old male diagnosed with stage III rectal adenocarcinoma treated with neoadjuvant chemoradiotherapy and curative surgery. Three years postoperatively, he developed a low-grade radiation-induced leiomyosarcoma in the perianal region. Histopathological examination confirmed a spindle cell neoplasm with notable immunohistochemical markers. RIS often presents as aggressive high-grade tumors resistant to radiotherapy and chemotherapy, necessitating surgical resection as the primary treatment. This case underscores the importance of long-term surveillance post-radiotherapy and highlights the need for innovative therapeutic strategies, including immunotherapy. Despite being rare, RIS poses a significant risk following cancer treatment, making early detection through vigilant monitoring and advancements in therapeutic approaches crucial for improving patient outcomes.

## 1 Introduction

Radiation-induced sarcomas (RIS) are iatrogenic malignancies that emerge as a result of high-dose radiation therapy administered during prior cancer treatments. Although RIS are relatively rare, constituting approximately 0.5%-5% of all sarcomas (1), their clinical importance is substantial. The incidence of RIS is expected to increase with the advancements in radiotherapy and its widespread use in oncology (2). The development of RIS presents significant health risks for patients and poses a considerable challenge for clinicians in selecting appropriate therapeutic strategies. Studies have shown that radiation-induced sarcomas predominantly include angiosarcomas, accounting for 48.1-57.9% of cases (3). These sarcomas are associated with poor prognosis and increased morbidity compared to primary sarcomas (4).Currently, RIS lacks a standardized definition, but the criteria commonly employed, proposed, and modified by Cahan’s criteria (5) include the appearance of a new tumor within the irradiated field, a substantial latency period between radiation exposure and tumor onset, and histopathological confirmation of sarcoma.

Radiation therapy has become an essential treatment modality for various malignancies, effectively utilizing high-energy radiation to eradicate cancer cells and significantly prolong patient survival. However, the widespread application of radiation therapy is not without potential complications. Rectal cancer, a common malignancy of the digestive system, often requires a multimodal approach to treatment, including surgery, chemotherapy, and radiation therapy. Incorporating radiation therapy into rectal cancer treatment protocols has been associated with improved survival rates and reduced local recurrence (6). Studies have demonstrated that radiation therapy enhances patient outcomes by decreasing the risk of local recurrence (7). Total neoadjuvant therapy (TNT) has emerged as a promising strategy in the management of rectal cancer. TNT involves administering radiation or chemoradiotherapy alongside systemic chemotherapy prior to surgery, aiming to increase treatment completion rates, reduce toxicity, optimize tumor response, and ultimately improve survival outcomes (8). Additionally, short-course radiation therapy has been introduced as a convenient and cost-effective alternative, eliminating the need for additional radiosensitizing chemotherapy (9). Innovative techniques such as hydrogel spacers, like SpaceOAR, have been explored to decrease rectal radiation exposure during prostate cancer treatment, underscoring the importance of minimizing treatment-related complications (10). Further research into the molecular mechanisms behind therapeutic radiation-induced radioresistance in rectal cancer aims to enhance the efficacy of radiotherapy and improve clinical outcomes.

Despite these advancements, radiation therapy for rectal cancer faces significant challenges. Resistance to radiation therapy remains a major clinical obstacle. Studies have indicated that pre-existing subclones can impact radioresistance in rectal cancer, highlighting the necessity for personalized treatment strategies to overcome resistance mechanisms (11). Additionally, ongoing investigations are evaluating the feasibility and toxicity of TNT combined with short-course radiation therapy followed by chemotherapy to optimize treatment outcomes for rectal cancer patients (12). The management of RIS poses a considerable challenge for clinicians in selecting appropriate therapeutic strategies due to the rarity and complexity of these malignancies (13). Despite the benefits of radiotherapy in cancer treatment, RIS represents a severe iatrogenic complication with poor survival outcomes (14, 15). The genetic signature of radiation-induced sarcomas has been found to be distinct, characterized by C-to-T mutations indicative of oxidative damage (16). The clinical diagnosis and management of RIS are challenging due to the prolonged latency period, which can lead to under-recognition of the condition. Diagnosis relies on comprehensive patient history, imaging studies, and histopathological examination. Treatment options for RIS are limited, with surgical resection being the primary modality, given the general resistance of RIS to radiochemotherapy. Unfortunately, the prognosis for RIS is typically worse than for primary sarcomas.

This report presents a case of a 71-year-old male diagnosed with stage III (T3N2M0) rectal adenocarcinoma in December 2018, who underwent neoadjuvant chemoradiotherapy followed by curative surgery. Three years postoperatively, the patient developed a low-grade radiation-induced leiomyosarcoma in the perianal region. Through this case, we aim to heighten clinical awareness of RIS, discuss diagnostic and therapeutic challenges, and provide insights that may inform future clinical practice and research efforts in radiation oncology.

## 2 Case presentation

### 2.1 Diagnosis, imaging, and treatment course

**Figure 1:**
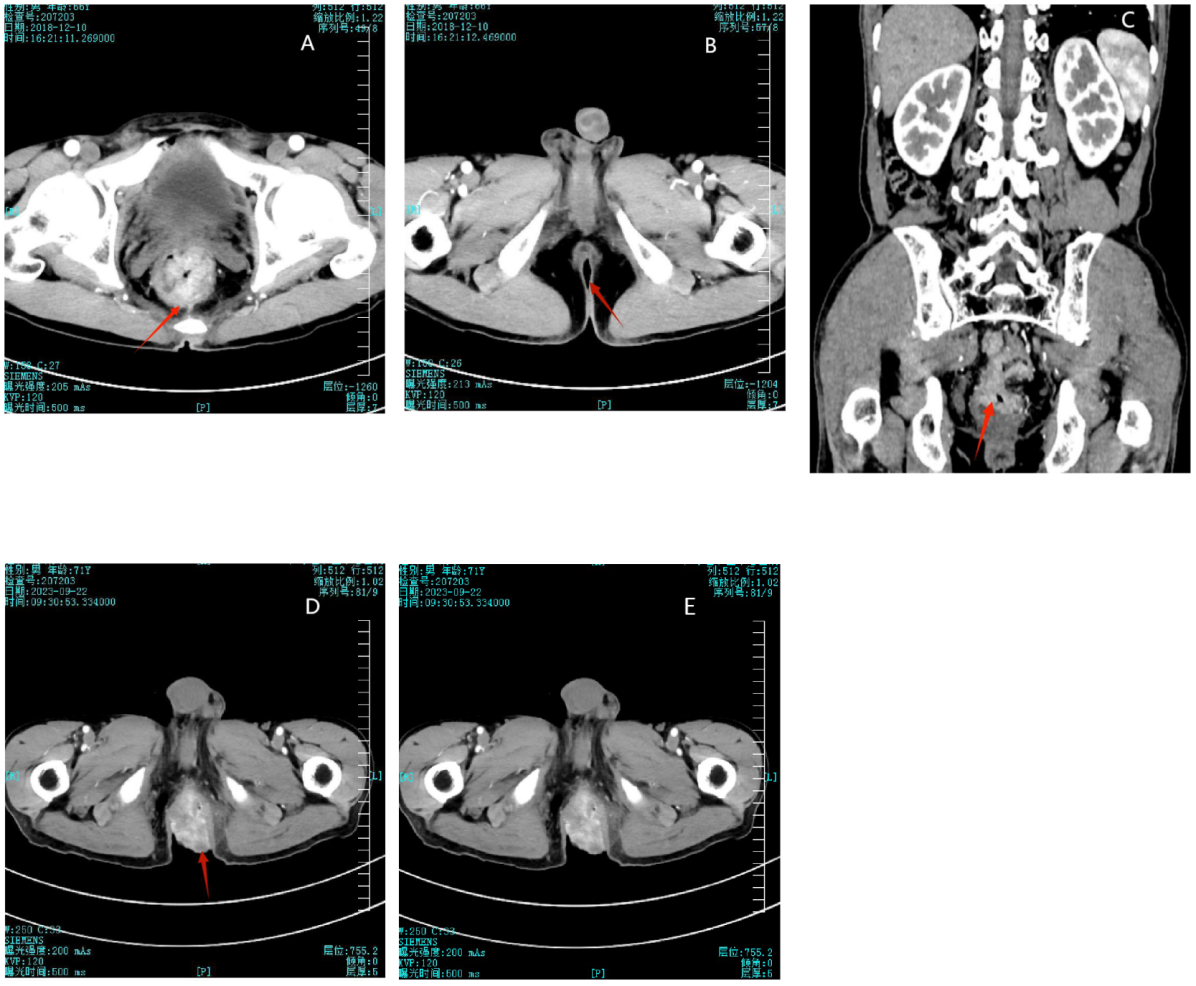
**(A)** Initial detection of rectal mass in 2018. **(B)** No abnormalities shown in perianal imaging in 2018. **(C)** 2018 imaging showing the rectal mass and normal perianal area. **(D)** Disappearance of the rectal mass after treatment in 2023. **(E)** The perianal mass did not decrease significantly after chemoradiotherapy.

The patient is a 71-year-old male who was diagnosed with stage III (T3N2M0) rectal adenocarcinoma in December 2018 (**Figure 1A**). From December 20, 2018, to January 24, 2019, he underwent neoadjuvant radiotherapy with a total dose of 50GY administered in 25 fractions (DT50GY/25F), concurrently with weekly oral capecitabine (Xeloda, Roche). During this period, imaging of the perianal region showed no abnormalities (**Figures 1B, C**). On March 11, 2019, the patient underwent a curative resection for rectal cancer (DIXON procedure). Postoperatively, from April to August 2019, he received three cycles of chemotherapy with oxaliplatin (Eloxatin, Sanofi) and capecitabine (XELOX) and an additional five cycles of capecitabine monotherapy.

A CT scan on September 22, 2023, demonstrated uniform thickening of the rectal wall, with a maximum thickness of 12 mm, and significant enhancement on contrast imaging. The perirectal fat spaces appeared normal, with no abnormal signal observed (**Figure 1D**).

Because the patient was elderly, lived alone, and had limited mobility, he faced significant challenges in seeking timely medical care. Without immediate family support due to his children’s work commitments in distant cities and constrained by financial difficulties, the patient did not promptly visit a healthcare provider after self-detecting a nodule in the perianal region in January 2022. Instead, he attempted to manage the condition by applying over-the-counter traditional Chinese medicine. However, the lesion gradually enlarged. It was not until June 2022, when his children returned and became aware of the worsening condition, that they arranged for the patient to seek medical evaluation. An abdominal MRI on June 2, 2022, revealed an irregularly shaped soft tissue mass in the perianal area, protruding outward and measuring approximately 57 mm × 51 mm. The mass exhibited heterogeneous signals and significant enhancement on contrast imaging, invading both levator ani muscles, and was considered a recurrence of the primary tumor. Pathological biopsy confirmed a diagnosis of a low-grade mesenchymal tumor. The pathologist recommended further immunohistochemistry and genetic testing; however, due to the patient’s financial difficulties, the family declined these additional investigations.

From June 9 to September 2, 2023, the patient received five cycles of chemotherapy consisting of capecitabine and irinotecan (Camptosar, Pfizer) plus bevacizumab (Avastin, Genentech). A CT scan performed on September 22, 2023, demonstrated uniform thickening of the rectal wall, with a maximum thickness of 12 mm, and significant enhancement on contrast imaging. The perirectal fat spaces appeared normal, with no abnormal signal observed, indicating that the rectal tumor bed mass had disappeared (**Figure 1D**). However, despite this treatment, the perianal mass showed no significant reduction in size (**Figures 1E**). Due to the lack of response to chemotherapy, the patient and their family expressed a loss of confidence in further curative treatment and considered transitioning to palliative care. The patient was discharged. In October 2023, the mass had increased in size, resulting in bowel obstruction and significant discomfort. This development led to the decision to pursue surgical intervention. The patient was readmitted, and on October 10, 2023, underwent resection of the rectal and perianal masses. The resected specimens were submitted for pathological examination. After the surgery, the patient was again discharged to receive palliative care. No further follow-up information was available, and in May 2024, the patient died due to disease progression.

### 2.2 Ethics statement

This study was approved by the Ethics Committee of the Central Hospital of Guangdong Provincial Nongken, Zhanjiang (Approval Number (20240715):).Written informed consent was obtained from the patient’s family for the publication of this case report and any accompanying images.

### 2.3 Pathological and imaging assessments

**Figure 2:**
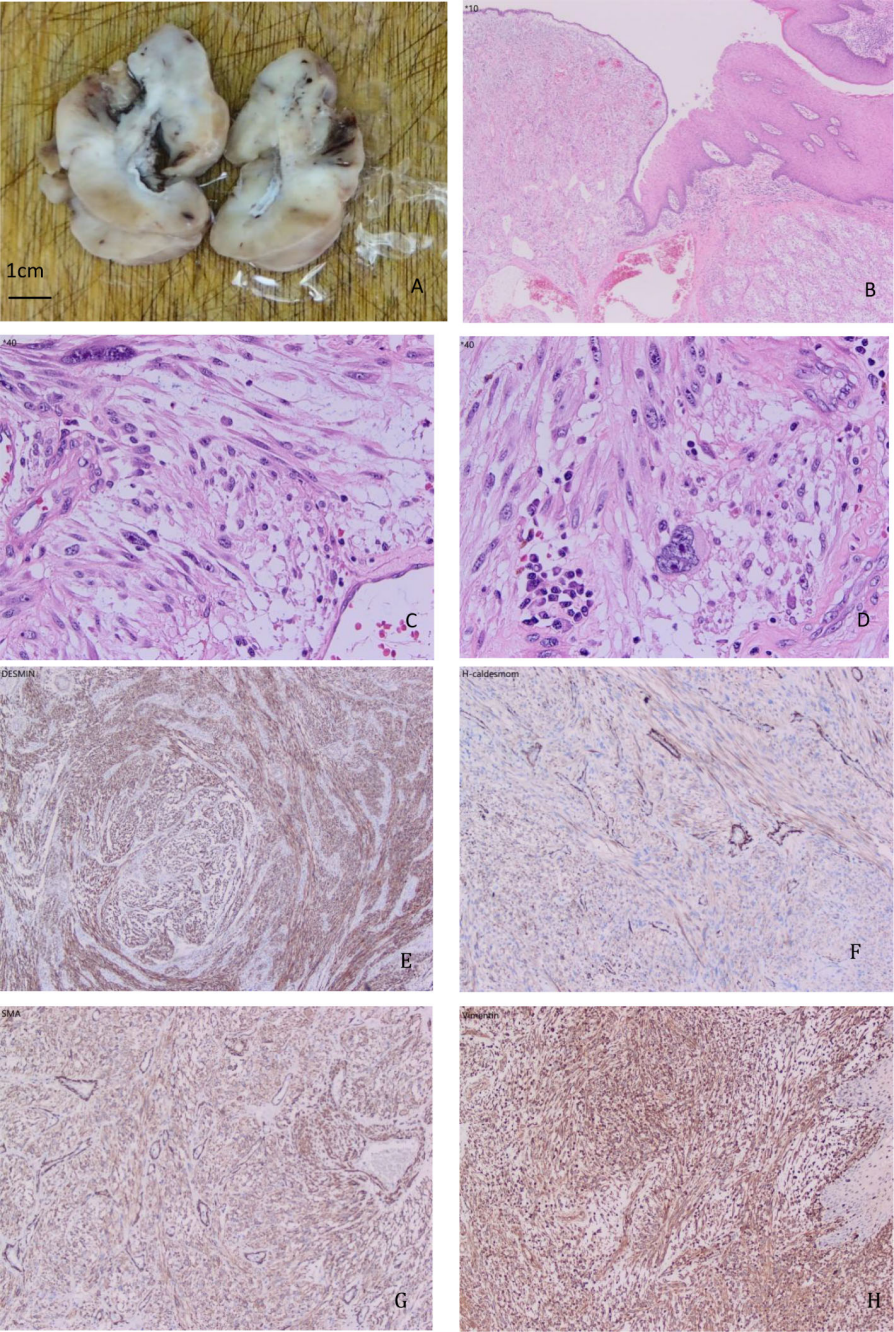
**(A)** Gross morphology of the tumor. **(B–D)** HE staining morphology (magnifications: **(B)** x50, **(C)** x200, **(D)** x200). **(E)** Desmin immunohistochemical staining (x100). **(F)** H-caldesmon immunohistochemical staining (x100). **(G)** SMA immunohistochemical staining (x100). **(H)** Vimentin immunohistochemical staining (x100).

Pathologic diagnosis revealed a spindle cell neoplasm. Based on the patient’s clinical history and immunohistochemical profile, the tumor was diagnosed as a radiation-induced low-grade leiomyosarcoma (**Figure 2**).

Microscopic examination demonstrated that the neoplasm was composed of spindle cells arranged in interlacing fascicles and bundles. The tumor cells exhibited abundant eosinophilic cytoplasm and centrally located nuclei. In some regions, irregularly shaped, hyperchromatic giant tumor cells with pronounced atypia were observed. Occasional tumor cell nuclei displayed vacuolation, resulting in nuclear indentations. Mitotic figures were infrequent, approximately 3-5 per 10 high-power fields. The stroma was richly vascularized, with tumor cells often arranged around blood vessels, resembling a pericytic growth pattern.

Immunohistochemical staining using the EnVision method revealed positivity for SMA, Vimentin, p16, p53 (40%), Ki-67 (20% in hotspots), Desmin, Calponin, SATB2 (focally), and H-Caldesmon. The tumor was negative for CD34, CK8/18, p40, CK-P, SOX10, Melan-A, Ep-CAM, ALK, and CD117. The positivity for SMA and Vimentin supported the tumor’s smooth muscle and mesenchymal origin, while the Ki-67 index of 20% in hotspots indicated moderate proliferative activity.

In conclusion, considering the microscopic morphologic characteristics, immunohistochemical profile, and the patient’s clinical history, the diagnosis was established as a radiation-induced low-grade leiomyosarcoma.

## 3 Discussion

The most common histological subtypes of radiation-induced sarcomas are osteosarcoma (21%), malignant fibrous histiocytoma (16%), and angiosarcoma/lymphangiosarcoma (15%) (17). RIS typically manifests as aggressive tumor subtypes and behaviors, with the majority being high-grade malignancies (2). They are generally resistant to radiotherapy and chemotherapy, making them difficult to cure and associated with a poorer prognosis compared to primary sarcomas. The pathogenesis of radiation-induced sarcomas (RIS) is multifaceted, primarily involving radiation-induced DNA damage and genomic instability. Radiation exerts its effects through direct and indirect mechanisms, leading to DNA double-strand breaks and genetic mutations. If these damages are not effectively repaired, genomic instability ensues, increasing the risk of tumorigenesis. Major risk factors include exposure to high-dose radiotherapy at a young age, concurrent chemotherapy with alkylating agents, and hereditary conditions such as Gardner syndrome and Li-Fraumeni syndrome (18, 19). These factors exacerbate DNA damage and genomic instability, thereby elevating the risk of RIS. The anatomical distribution of radiation-induced tumors varies, likely due to the differential application of radiotherapy across anatomical sites. For instance, radiotherapy is more frequently administered to the head and neck region, resulting in a higher incidence of radiation-induced tumors in this area. In contrast, the lower incidence of such tumors in the lower digestive tract can be attributed to the reduced necessity for radiotherapy in this region. This discrepancy underscores the critical importance of vigilant monitoring of lesions within the radiation target area post-radiotherapy.

The diagnosis of RIS necessitates a meticulous medical history, comprehensive imaging studies, and histopathological confirmation. The protracted latency period of RIS, often manifesting several years to decades post-radiotherapy, poses a significant diagnostic challenge, as clinicians may not immediately associate new malignancies with prior radiotherapy. Reports indicate that the median latency period for radiation-induced sarcomas is approximately 10 years, with a range extending from 6 months to 20 years. There are instances of cutaneous angiosarcomas emerging as early as 4 years following radiotherapy (20). This extensive latency period complicates the early detection of RIS. Furthermore, the histological resemblance between RIS and primary sarcomas further complicates the diagnostic process. RIS often presents as highly aggressive tumors, with histological characteristics that may overlap with those of primary sarcomas, necessitating the use of immunohistochemical staining and molecular markers for accurate differentiation.

At present, surgical resection remains the cornerstone of treatment for RIS. However, due to the intrinsic resistance of RIS to conventional radiotherapy and chemotherapy, the prognosis is typically poorer compared to primary sarcomas. Research indicates that radiation-induced sarcomas are often characterized by higher invasiveness and recurrence rates, making surgical intervention the primary treatment modality, albeit with a higher risk of postoperative complications, particularly in lower extremity sarcomas (21). Treatment planning for RIS must incorporate a comprehensive assessment of surgical risks and the patient’s overall health status. Despite the limitations of traditional therapeutic approaches, recent studies have demonstrated the potential efficacy of PD-1 blockade immunotherapy for RIS patients. These novel therapies, by augmenting the immune response, hold promise for improving the prognosis of RIS patients (22). Emerging evidence suggests that the immune system plays a critical role in the development and progression of radiation-induced sarcomas (RIS) (23). Radiation not only induces DNA damage but also alters the tumor microenvironment, leading to immune suppression and facilitating tumor escape from immune surveillance. Immunotherapy, particularly immune checkpoint inhibitors such as PD-1/PD-L1 inhibitors, has shown promise in overcoming these immune evasion mechanisms. By reinvigorating exhausted T cells and restoring immune function, these agents may enhance the immune system’s ability to recognize and eliminate radiation-induced tumor cells. Preclinical and early clinical studies indicate that combining radiotherapy with immunotherapy may have synergistic effects, improving tumor control and potentially reducing the risk of recurrence (24). Further research is needed to fully elucidate the immunological mechanisms involved in RIS and to optimize the use of immunotherapy in this context. Exploring the role of immune biomarkers in predicting response to therapy could also facilitate the development of more personalized treatment strategies for patients with radiation-induced sarcomas. It is imperative to understand the mechanisms of radiotherapy-induced injuries within the digestive system to ensure accurate diagnosis, effective treatment, and the minimization of treatment-related complications. Long-term surveillance and follow-up of patients who have undergone radiotherapy, particularly those involving high-risk regions, are essential for the early identification and management of radiation-induced complications, including sarcomas.

The patient initially presented with stage III (T3N2M0) rectal adenocarcinoma and underwent neoadjuvant chemoradiotherapy followed by curative surgery. Three years postoperatively, a low-grade radiation-induced leiomyosarcoma was identified in the perianal region. Microscopic examination revealed sparse mitotic figures (3-5/10 high-power fields), no significant necrosis, and marked cellular atypia. Immunohistochemical staining showed positivity for SMA, Vimentin, p16, p53, Ki-67, Desmin, Calponin, SATB2, and H-Caldesmon, while CD34, CK8/18, p40, CK-P, SOX10, Melan-A, Ep-CAM, ALK, and CD117 were negative. Based on the clinical presentation and immunohistochemical profile, differential diagnoses such as inflammatory myofibroblastic tumor, pseudomyogenic hemangioendothelioma, and fibrosarcoma were excluded, confirming the diagnosis of low-grade leiomyosarcoma.

Primary leiomyosarcoma of the rectum is exceedingly rare, constituting less than 0.1% of colorectal tumors (25). No more than 290 cases have been reported in the literature (26). Radiation-induced leiomyosarcoma following rectal cancer radiotherapy is even rarer, with few cases documented in the literature. One such case involved rectal leiomyosarcoma occurring 32 years after cervical cancer radiotherapy (27). In contrast, the latency period in our case was relatively short, at only 3 years. Furthermore, the tumor exhibited low mitotic activity and a low Ki-67 index, possibly indicative of an early stage of tumor development. Despite this, our case underscores the potential risk of radiation-induced tumors in the lower gastrointestinal tract as a consequence of radiotherapy.

While radiotherapy is a cornerstone of cancer treatment, its potential to induce secondary malignancies, such as sarcomas, cannot be overlooked. This case emphasizes the necessity of long-term surveillance of irradiated fields following radiotherapy to identify and manage potential radiation-induced complications promptly. Enhanced monitoring is warranted for patients who have received high-dose radiotherapy, focusing on high-risk areas. The management of this case provides valuable insights, highlighting the importance of early diagnosis through thorough medical history, regular imaging studies, and histopathological evaluation. Given the general resistance of radiation-induced sarcomas to conventional radiotherapy and chemotherapy, surgical resection remains the primary treatment modality. However, personalized treatment plans are essential based on individual patient circumstances. Future research should aim to elucidate the mechanisms underlying radiation-induced sarcomas and explore novel therapeutic strategies, including immunotherapy and targeted therapy (28).

Additionally, educating clinicians and patients about the potential risks of radiotherapy is crucial to promote early detection and intervention. This case not only enriches the clinical understanding of radiation-induced leiomyosarcoma but also offers important considerations for future clinical practice.

This case report has certain limitations. Genetic testing was not pursued due to the patient’s financial limitations, which precluded a deeper molecular analysis of the tumor and the identification of potential therapeutic targets. Furthermore, the lack of extended follow-up after the patient’s discharge to palliative care resulted in an absence of data regarding surgical margin status, postoperative morbidity, and sphincter function. In addition, no photographic documentation was obtained before, during, or after the surgical procedure, limiting the objective assessment of surgical findings and outcomes.

## 4 Conclusion

Radiation-induced sarcomas (RIS) represent a significant yet rare complication of cancer treatment, with its clinical importance underscored by the complexity of its pathogenesis. RIS involves mechanisms such as radiation-induced DNA damage and genomic instability. Key risk factors include receiving high-dose radiotherapy at a young age, concurrent chemotherapy with alkylating agents, and hereditary conditions such as Gardner syndrome and Li-Fraumeni syndrome. Currently, the diagnosis of RIS relies on comprehensive patient history, imaging studies, and histopathological confirmation. However, the long latency period of RIS often leads to under-recognition by clinicians during treatment. Additionally, the histological similarity between RIS and primary sarcomas complicates the diagnosis. Despite surgical resection being the primary treatment modality, RIS generally shows a poor prognosis compared to primary sarcomas due to its resistance to radiotherapy and chemotherapy.

Future research should focus on elucidating the molecular mechanisms of radiation-induced carcinogenesis, particularly genomic instability and DNA repair pathways, to develop new preventive and therapeutic strategies. Furthermore, developing more sensitive and specific diagnostic tools, incorporating imaging and molecular markers, could facilitate early detection of RIS and improve diagnostic accuracy. Exploring innovative treatment methods, including immunotherapy and targeted therapy, alongside existing treatment modalities will be crucial in improving the prognosis for RIS patients.

Clinical monitoring and patient education are pivotal in the early detection of RIS. Long-term follow-up and monitoring of patients who have undergone radiotherapy, especially those at high risk, are essential for the timely identification and management of potential RIS. Increasing awareness among clinicians and patients about RIS and the potential risks associated with radiotherapy will promote early detection and intervention. Establishing multidisciplinary collaboration and integrating expertise from oncology, radiology, pathology, and genetics to provide personalized management plans for RIS will also contribute to better patient outcomes.

In conclusion, although radiation-induced sarcomas are rare, they significantly impact patient health and therapeutic decisions. By advancing research on RIS, improving early detection capabilities, and exploring innovative treatment approaches, we can enhance the prognosis for RIS patients and further the field of radiation oncology.

## Data Availability

The original contributions presented in the study are included in the article/supplementary material. Further inquiries can be directed to the corresponding author.
